# Platelet-Rich Plasma for Wound Healing and Scar Outcomes After Cesarean Section: A Systematic Review and Meta-Analysis

**DOI:** 10.3390/healthcare14142108

**Published:** 2026-07-14

**Authors:** Ana-Maria Brezeanu, Dragos Brezeanu, Vlad-Iustin Tica

**Affiliations:** 1 6th Department, Faculty of Medicine, Ovidius University of Constanta, 900527 Constanta, Romania; anmariataras@gmail.com (A.-M.B.); vtica@eeirh.org (V.-I.T.); 2 County Clinical Emergency Hospital “Sf. Ap. Andrei”, 900500 Constanta, Romania; 3 Romanian Academy of Scientists, 50444 Bucharest, Romania

**Keywords:** platelet-rich plasma, cesarean section, wound healing, scar, REEDA, POSAS, uterine niche, systematic review, meta-analysis, PRISMA 2020, platelet concentrate

## Abstract

**Background:** Cesarean section (CS) is one of the most frequently performed surgical procedures worldwide, with postoperative wound complications and suboptimal scarring contributing to maternal morbidity. Platelet-rich plasma (PRP) has been proposed as a regenerative adjunct; however, existing evidence remains heterogeneous and incompletely synthesized. **Objectives**: The purpose of this review was to evaluate the efficacy and safety of PRP for wound healing and scar outcomes after cesarean section, including uterine scar healing outcome **Methods:** This systematic review and meta-analysis was conducted according to PRISMA 2020 guidelines and prospectively registered in PROSPERO (CRD420261383413). Major databases were searched from inception to April 2026 for randomized controlled trials (RCTs) evaluating PRP versus standard care in women undergoing CS. Primary outcomes included scar quality (POSAS) and early wound healing (REEDA), while secondary outcomes included pain (VAS), Vancouver Scar Scale (VSS), and uterine scar parameters. Random-effects models were used, and risk of bias and certainty of evidence were assessed using established tools. **Results:** Five RCTs (*n* = 366) were included in the quantitative synthesis. PRP was associated with improved patient-reported scar quality (POSAS-patient: SMD −0.50, 95% CI −0.83 to −0.17) and clinician-assessed scar quality (POSAS-observer: SMD −0.42, 95% CI −0.75 to −0.10), as well as reduced early wound inflammation (REEDA: SMD −0.52, 95% CI −0.84 to −0.20), with low statistical heterogeneity. No significant reduction in early postoperative pain was observed (VAS: SMD −0.22, 95% CI −0.50 to 0.06). Evidence regarding uterine scar outcomes was limited and imprecise. No PRP-related adverse events were reported. **Conclusions:** Intraoperative PRP may improve scar quality and early wound healing following cesarean section while not significantly affecting early postoperative pain. Given the limited number of RCTs and variability in PRP protocols, these findings should be interpreted with caution. Further well-designed, adequately powered trials with standardized methodologies are required to confirm clinical effectiveness and long-term outcomes.

## 1. Introduction

Cesarean section (CS) accounts for approximately 21% of all births worldwide, a proportion projected to reach 28.5% by 2030 [1]. In Romania, national CS rates exceed 45%, with regional rates approaching 52% in certain demographic subgroups [2,3]. Wound complications—infection, dehiscence, seroma, and hematoma—affect 3–15% of patients, with substantially higher rates in women with obesity, diabetes mellitus, or multiple prior CS [4,5]. In high-risk populations such as women with class III obesity, wound breakdown risk is two- to fourfold higher, and women undergoing repeat CS operate through progressively less well-vascularized scar tissue [5].

Beyond immediate wound morbidity, suboptimal scar formation represents an under-recognized source of long-term physical and psychosocial burden, including chronic pain, impaired body image, and reduced sexual satisfaction [6,7]. A further dimension of cesarean wound healing—largely ignored in the surgical literature—is the uterine scar. Between one-third and two-thirds of women develop an isthmocele (hypoechoic myometrial defect at the uterotomy site) within the first year after a first CS [8]. This niche causes postmenstrual spotting, dysmenorrhea, and chronic pelvic pain and represents the structural substrate for cesarean scar ectopic pregnancy and for placenta accreta spectrum in subsequent pregnancies [8]. An intervention at the time of the index operation that reduces niche formation would carry consequences well beyond the immediate hospitalization.

Platelet-rich plasma (PRP) is derived from a small volume of the patient’s own blood by centrifugation, yielding a fraction enriched two- to fivefold in platelets. Upon activation, platelets release platelet-derived growth factor (PDGF), transforming growth factor-beta 1 and 2 (TGF-β1/2), vascular endothelial growth factor (VEGF), epidermal growth factor (EGF), fibroblast growth factor (FGF), and insulin-like growth factor-1 and -2 (IGF-1/2) in a coordinated surge that shortens the inflammatory phase, drives fibroblast proliferation and collagen synthesis, and supports angiogenesis in granulation tissue [9,10]. PRP has demonstrated efficacy across a broad spectrum of surgical applications [11,12,13]. Cord blood PRP (CB-PRP), collected from the umbilical vessels after delivery, is uniquely available only at cesarean and delivers a growth factor payload that exceeds adult peripheral blood by virtue of fetal hematopoietic biology [14].

Several RCTs have examined PRP in CS wound healing over the past decade. However, the evidence base has recently been materially affected by the formal retraction of Elkhouly et al. (2021)—Gynecol Obstet Invest 86(4):336–342—by Karger Publishers in May 2025 (PMID: 40435965), following an Expression of Concern issued in November 2021. This study had been one of the most frequently cited publications in this area, and its retraction necessitates a transparent re-evaluation of the corrected evidence base. Furthermore, existing reviews have not comprehensively evaluated the full spectrum of validated assessment instruments (including Manchester Scar Scale and Numeric Rating Scale), the CB-PRP versus autologous comparison, or the uterine scar dimension [15].

The present systematic review and meta-analysis was designed to (1) synthesize RCT evidence on PRP for post-cesarean wound healing using rigorous meta-analytic methods including dispersion-measure verification; (2) address the impact of the confirmed retraction; (3) explore the uterine scar as a distinct outcome domain; and (4) provide a GRADE-rated evidence summary to guide clinical decision making and future research priorities. The review is distinguished from a related concurrent review (Sen et al., INPLASY202640041, October 2025 [15]) by its prospective PROSPERO registration, broader outcome scope (MSS, NRS, and uterine scar), inclusion of the Kamel 2018 diabetic-population subgroup, explicit retraction documentation, and the team’s firsthand clinical data from the multidimensional Brezeanu 2025 series [16,17,18,19,20,21,22]. The primary endpoints of this review were scar quality assessed by POSAS (patient and observer components) and early wound healing assessed by the REEDA scale. Secondary endpoints were postoperative pain (VAS/NRS), Vancouver Scar Scale (VSS), Manchester Scar Scale (MSS), uterine scar parameters (niche formation and residual myometrial thickness), and adverse events [17,18,19,20,21,22].

## 2. Materials and Methods

This systematic review and meta-analysis were conducted and reported in accordance with the Preferred Reporting Items for Systematic Reviews and Meta-Analyses (PRISMA) 2020 guidelines [16,17] and registered prospectively in PROSPERO (CRD420261383413, April 2026). The review protocol was developed a priori, and no major deviations from the registered protocol occurred during the conduct of the review.

### 2.1. Eligibility Criteria

Studies were eligible if they met the following PICO criteria:

Population (P): Adult women (≥18 years) undergoing elective or emergency CS, any indication, gestational age, BMI, or number of prior CS.

Intervention (I): PRP of any type (autologous, leukocyte-poor/LP-PRP, or cord blood/CB-PRP), any preparation protocol, any activation status, any volume, and any administration route (local wound application, subcutaneous injection, or intramyometrial injection), administered intraoperatively or perioperatively.

Comparator (C): Standard wound care without PRP or placebo (saline solution).

Outcomes (O): At least one quantifiable outcome related to wound healing or scar quality (REEDA, POSAS, VSS, MSS, VAS, NRS, ultrasonography, wound complication rates, or uterine scar parameters).

Study designs: RCTs (primary meta-analysis) or non-randomized controlled trials and controlled observational studies (secondary narrative analysis); minimum 10 participants per group.

Exclusion criteria: (1) Studies evaluating exclusively internal uterine wound healing without any cutaneous scar outcome (unless reporting uterine niche as a distinct pre-specified outcome); (2) single-arm studies without a comparator (narrative only); (3) case reports/series < 10 participants per group; (4) animal or in vitro studies; (5) reviews, editorials, or letters; (6) retracted publications and their corresponding registry entries (specifically Elkhouly et al. 2021, PMID 34261076, retracted May 2025, and NCT04669353); and (7) studies where full-text data are unavailable and authors do not respond to data requests within 4 weeks.

### 2.2. Information Sources and Search Strategy

Five databases were searched from inception without date or language restrictions: PubMed/MEDLINE, Cochrane CENTRAL, Embase, Web of Science Core Collection, and Scopus. ClinicalTrials.gov and WHO ICTRP were searched for registered and unpublished trials. The final search was executed April 2026 with an update planned prior to submission. Full database-specific search strings are available in the supplementary search strategy document (uploaded to PROSPERO).

The core search string combined (1) PRP terminology: (“platelet-rich plasma” OR “PRP” OR “platelet rich plasma” OR “LP-PRP” OR “leukocyte-poor PRP” OR “cord blood PRP” OR “CB-PRP”); AND (2) population: (“cesarean section” OR “caesarean section” OR “C-section” OR “cesarean delivery” OR “caesarean delivery”); AND (3) outcome: (“wound healing” OR “scar” OR “scar healing” OR “wound” OR “postoperative healing” OR “uterine niche” OR “isthmocele”). Database-specific adaptations used MeSH (PubMed), Emtree (Embase), and controlled vocabulary for each platform.

### 2.3. Study Selection

Records were managed in Covidence. After automated deduplication, two reviewers (A-M.B. and D.B.) independently screened titles/abstracts then full texts. Disagreements were resolved by consensus and arbitration by V-I.T. Reasons for exclusion at the full-text stage were documented as per PRISMA requirements. To minimize investigator bias arising from the inclusion of author-affiliated studies, screening, full-text eligibility assessment, data extraction, and risk-of-bias evaluation for the two Brezeanu studies were performed by an independent external reviewer (an academic methodologist with no involvement in the primary studies and no co-authorship on this review) [22,23]. The review-team authors were excluded from all eligibility and extraction decisions concerning their own studies; data values were extracted directly from the published Healthcare articles and cross-checked against the published tables.

### 2.4. Data Extraction

Two reviewers independently extracted data using a standardized pre-piloted form capturing study and participant characteristics; PRP type and preparation protocol (centrifugation parameters, activation status, and volume); administration route and timing; and all pre-specified outcome data (means, standard deviations or standard errors, event counts, and *p*-values) at each timepoint. When dispersion-measure labeling was ambiguous, data were verified against reported *p*-values by back-calculation. Corresponding authors were contacted for missing data.

### 2.5. Risk-of-Bias Assessment

RCTs were assessed using the Cochrane Risk of Bias 2.0 tool (RoB 2) across five domains: (D1) randomization process; (D2) deviations from intended interventions; (D3) missing outcome data; (D4) outcome measurement; and (D5) selection of reported results. The observational study (Brezeanu et al. Study 2) was assessed using ROBINS-I [23]. Given the declared conflict of interest, RoB for Brezeanu 2025 studies was performed by an independent external reviewer. Two reviewers assessed all other studies independently; disagreements were resolved by consensus [22].

### 2.6. Statistical Analysis and Data Synthesis

Meta-analysis was performed for outcomes reported by at least two RCTs. Continuous outcomes were expressed as the Standardized Mean Difference (SMD; Hedges’ g) or Mean Difference (MD) with 95% confidence intervals using DerSimonian–Laird random-effects models. Negative SMD denotes PRP favored. Dichotomous outcomes were expressed as the Risk Ratio (RR) with the 95% CI. Statistical heterogeneity was quantified using I^2^ (substantial threshold: ≥50%) and Cochran’s Q test. Where I^2^ ≥ 75%, pooling was considered inappropriate, and narrative synthesis was performed.

Sensitivity analyses (pre-specified): (1) Exclusion of studies at high/some concerns of risk of bias; (2) exclusion of studies sharing authorship with the review team; (3) exclusion of CB-PRP study (Thanachaiviwat 2024); and (4) naive SD interpretation of Thanachaiviwat 2024 dispersion values (see Section 3.3). Subgroup analyses: PRP type (autologous vs. LP-PRP vs. CB-PRP); activation status; and administration route [21]. Publication bias: Funnel plot and Egger’s test where ≥5 studies contributed. Evidence certainty was rated using GRADE for each outcome; the results are presented in Summary of Findings (SoF) tables generated using GRADEpro GDT. Analyses were conducted using RevMan 5.4 and R 4.3.2 (metafor, meta packages).

## 3. Results

### 3.1. Study Selection: PRISMA Flow

**Figure 1:**
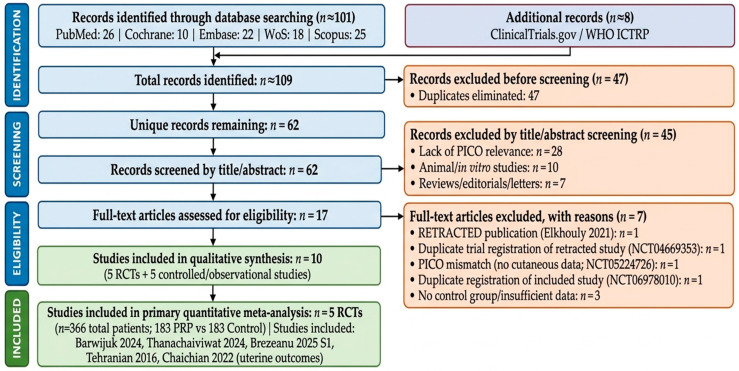
PRISMA 2020 flow diagram illustrating the study identification, screening, eligibility assessment, and inclusion process [17,18,20,21,22].

Database searches yielded approximately 101 records (PubMed = 26, Cochrane = 10, Embase = 22, WoS = 18, and Scopus = 25), with 8 additional records from ClinicalTrials.gov and WHO ICTRP (total *n* ≈ 109). After automated duplicate removal (47 duplicates), 62 unique records were screened by title/abstract; 45 were excluded. Seventeen full texts were assessed for eligibility; seven were excluded with documented reasons (PRISMA flow, Figure 1). Ten studies were included in qualitative synthesis; five RCTs were included in primary quantitative meta-analysis (*n* = 366). Throughout this review, study counts are reported consistently as follows: 10 studies in qualitative synthesis and 5 RCTs in the primary quantitative meta-analysis (*n* = 366), with Chaichian 2022 contributing to the uterine scar narrative analysis only and not to the cutaneous scar meta-analysis [18].

### 3.2. Study Characteristics

Table 1 presents the characteristics of all 10 included studies. Five RCTs constituting the primary meta-analysis pool enrolled a total of 366 women (183 PRP and 183 control) across five countries (Iran, Poland, Egypt, Thailand, and Romania) over 2016–2025. Two important corrections from preliminary extraction are noted. First, Tehranian 2016 [17] enrolled *n* = 67 PRP and *n* = 71 control (total *n* = 138), not the *n* = 30/30 attributed in several secondary citations. Second, Chaichian 2022 [18] enrolled *n* = 15 per group and evaluated the uterine scar, not the abdominal skin: PRP was injected between the decidua and myometrium at hysterorrhaphy closure, and the primary outcomes were niche formation rate and residual myometrial thickness at 6 months. Chaichian 2022 is retained in the systematic review and contributes uterine scar data but is excluded from the skin scar quantitative synthesis (see Section 3.5) [18].

### 3.3. Risk-of-Bias Assessment

To evaluate the internal validity of the included evidence, the risk of bias was assessed using the Cochrane RoB 2.0 tool for randomized controlled trials and ROBINS-I for the observational study. The overall assessments are presented in Table 2.

### 3.4. Quantitative Meta-Analysis: Primary and Secondary Outcomes

#### 3.4.1. Scar Quality: POSAS (Patient and Observer)

Two RCTs contributed POSAS data: Barwijuk 2024 (*n* = 23/23, day 90) and Brezeanu 2025 S1 (*n* = 50/50, day 40) [20,22]. Both studies showed consistent directional benefit of PRP on scar quality despite differing populations, PRP preparations, and assessment timepoints.

**Figure 2:**
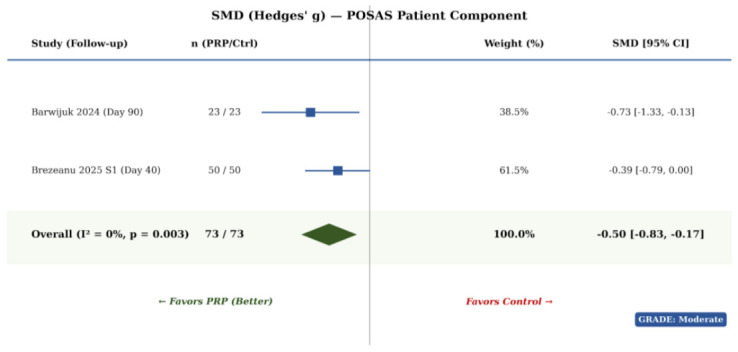
Forest plot of the meta-analysis comparing PRP vs. control for scar quality (POSAS Patient Component) [20,22].

POSAS-patient, day 90 (Barwijuk): 14.91 ± 1.54 vs. 16.09 ± 1.68; SMD −0.73 (95% CI −1.33 to −0.13). POSAS-patient, day 40 (Brezeanu): 7.24 ± 1.81 vs. 8.00 ± 2.06; SMD −0.39 (95% CI −0.79 to 0.00). The absolute scores differ because the studies use different POSAS total-score conventions; SMD pooling is appropriate. Pooled POSAS-patient: SMD −0.50 (95% CI −0.83 to −0.17; *p* = 0.003; I^2^ = 0%; Figure 2). GRADE: Moderate [20,22].

**Figure 3:**
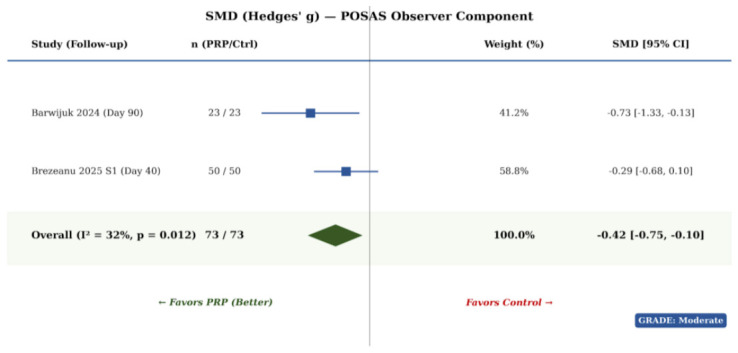
Forest plot of the meta-analysis comparing PRP vs. control for scar quality (POSAS Observer Component) [20,22].

POSAS-observer, day 90 (Barwijuk): 13.39 ± 1.53 vs. 14.74 ± 2.11; SMD −0.73. POSAS-observer, day 40 (Brezeanu): 6.46 ± 1.23 vs. 6.84 ± 1.39; SMD −0.29 [20,22]. The smaller clinician effect in Brezeanu at day 40 is biologically coherent as inflammatory signs dominate observer assessment and resolve more slowly [20,22]. Pooled POSAS-observer: SMD −0.42 (95% CI −0.75 to −0.10; *p* = 0.012; I^2^ = 32%; Figure 3). GRADE: Moderate.

#### 3.4.2. Early Wound Inflammation: REEDA Scale

**Figure 4:**
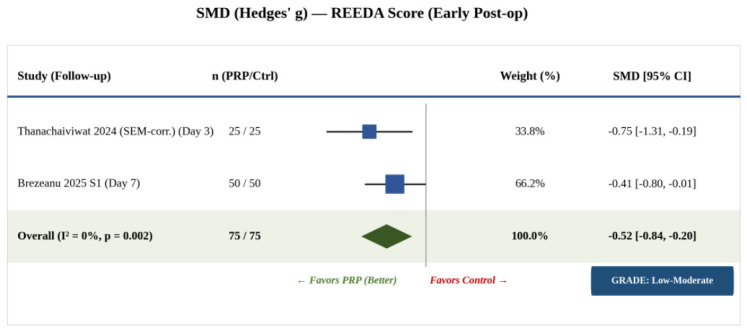
Forest plot of the meta-analysis for REEDA Score (Early Post-Op.): PRP versus Control [21,22].

Two RCTs contributed early REEDA data after SEM correction of Thanachaiviwat 2024 [21]. Thanachaiviwat 2024 (CB-PRP, day 1, SEM-corrected): 1.50 vs. 2.50; SMD −0.75 (95% CI −1.31 to −0.19) [21]. Brezeanu 2025 S1 (LP-PRP, day 7): 0.88 ± 1.09 vs. 1.56 ± 2.10; SMD −0.41 (95% CI −0.80 to −0.01) [22]. Pooled REEDA: SMD −0.52 (95% CI −0.84 to −0.20; *p* = 0.002; I^2^ = 0%; Figure 4). Sensitivity analysis with naive SD interpretation: Thanachaiviwat individual SMD becomes −3.82, producing I^2^ = 98%; pooling is inappropriate under this assumption [21,22]. The primary analysis result is conditional on the SEM identification being correct, which the back-calculation confirms. GRADE: Low–Moderate. Although back-calculation supports SEM interpretation, this remains a potential source of uncertainty affecting pooled estimates.

#### 3.4.3. Postoperative Pain: VAS

Key Finding: VAS Pain is NOT Significantly Reduced

**Figure 5:**
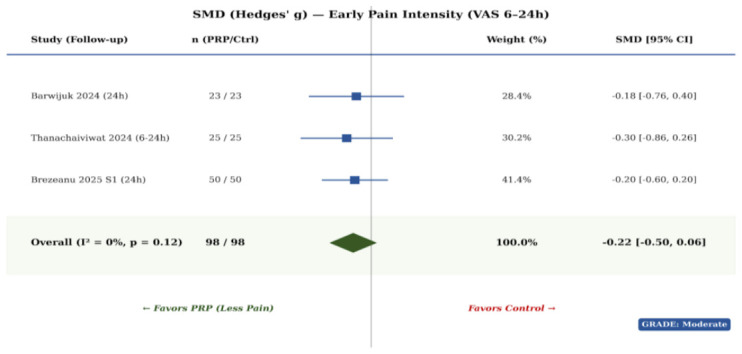
Forest plot of the meta-analysis for pain intensity VAS (Early Post-Op.): PRP versus Control [20,21,22,23].

Three RCTs contributed VAS data (Barwijuk 2024, Thanachaiviwat 2024, and Brezeanu 2025 S1) [20,21,22,23]. Pooled SMD = −0.22 (95% CI −0.50 to +0.06; *p* = 0.12; I^2^ = 0%, Figure 5). PRP does not significantly reduce early pain intensity at 6–24 h. This finding is robust (I^2^ = 0%) and unaffected by any sensitivity analysis. It is clinically coherent: PRP accelerates structural tissue reorganization rather than modulating the acute nociceptive signal. However, analgesic consumption data tell a different story: Barwijuk 2024 reports fewer paracetamol doses/day in the PRP arm (*p* = 0.006) and fewer supplemental opioid/NSAID requirements, consistent with a delayed analgesic effect [20]. Tehranian 2016 reported a 93% vs. 79% cumulative VAS reduction at 8 weeks (*p* < 0.001). GRADE: Moderate (for pain non-reduction) [17].

#### 3.4.4. Scar Quality: Vancouver Scar Scale (VSS): High Heterogeneity

Two studies contributed VSS data. Thanachaiviwat 2024 (CB-PRP, week 8): 2.58 ± 2.00 vs. 6.96 ± 2.44; SMD −1.97 (95% CI −2.63 to −1.30; *p* < 0.001) [21]. Brezeanu 2025 S1 (LP-PRP, day 40): 0.46 ± 0.73 vs. 0.76 ± 1.18; SMD −0.31 (95% CI −0.70 to +0.09; *p* = NS). I^2^ = 94%; Q = 17.8 (*p* < 0.001) [22]. These two studies cannot be pooled; the heterogeneity is substantial and likely reflects genuine differences between CB-PRP (higher growth factor payload, week 8 assessment) and LP-PRP (day 40 assessment). Narrative synthesis only. GRADE: Low [21,22].

#### 3.4.5. Uterine Scar: Niche Formation

Chaichian 2022 is the only RCT to evaluate the uterine scar as a primary endpoint [18]. PRP was injected between decidua and myometrium at uterotomy closure in 15 women vs. 15 controls. At 6-month transvaginal ultrasound, niche formation = 2/15 (13.3%) vs. 7/15 (46.7%); RR = 0.29 (95% CI 0.07–1.16); and Fisher’s exact *p* = 0.109 (two-sided). The study-reported *p* = 0.002 cannot be reproduced from the published 2 × 2 table and almost certainly derives from a continuous endpoint (niche depth, height, or residual myometrial thickness) rather than the binary rate. The binary result does not reach conventional statistical significance; the absolute difference (33 percentage points) and direction are consistent with the biological hypothesis. The clinical context is compelling: niche formation at 47% in the control arm is not an artefact; it is the clinical reality of the cesarean scar, and downstream consequences (isthmocele, ectopic implantation, and placenta accreta spectrum) are consequential enough to warrant a properly powered confirmation trial. GRADE: Low.

#### 3.4.6. Extended Outcomes: MSS, NRS, and Hematological Parameters

The observational arm (Brezeanu 2025 S2, *n* = 50) provided the first application of the Manchester Scar Scale (MSS) and Numeric Rating Scale (NRS) in this field, alongside serial hematological monitoring [23]. LP-PRP was administered via a standardized biphasic protocol: 20 mL blood collected after spinal anesthesia, with two-spin centrifugation (1600 rpm × 10 min; 3500 rpm × 7 min; XC Spin Plus centrifuge, XCmed, Bucharest, Romania), yielding 6–10 mL LP-PRP; 5 mL along uterine incision margins before hysterorrhaphy + 5 mL subcutaneously before skin closure. All six scar and pain scales improved significantly between Day 7 and Day 40 as presented in Table 3.

### 3.5. Complete Extracted Outcome Data

For each study, outcome measures, assessment timepoints, descriptive statistics, statistical significance, and corresponding effect sizes are presented. Where necessary, reported summary statistics were converted to a common format to enable quantitative synthesis. The main outcome data extracted from the included studies are summarized in Table 4.

### 3.6. Certainty of Evidence (GRADE Assessment)

The certainty of evidence for each outcome was assessed using the GRADE approach. A Summary of Findings table is presented in Table 5. The GRADE assessment and footnote justifications were performed in accordance with standard GRADE recommendations for transparency and reproducibility.

Overall, the certainty of evidence ranged from low to moderate. Moderate-certainty evidence supports the beneficial effect of PRP on patient- and observer-reported scar quality (POSAS). Early wound healing (REEDA) showed a similar effect size but with lower certainty due to methodological concerns and limited sample size. No significant reduction in early postoperative pain (VAS) was observed, supported by moderate-certainty evidence. Evidence for uterine scar outcomes remains limited and of low certainty. No adverse events related to PRP were reported; however, the certainty of safety data remains limited due to small sample sizes.

## 4. Discussion

### 4.1. Principal Findings

This systematic review and meta-analysis, conducted in full accordance with PRISMA 2020 guidelines, synthesizes evidence from five eligible RCTs spanning five countries: Tehranian et al. (2016, Iran, high-risk population), Chaichian et al. (2022, Iran, intramyometrial PRP), Kamel HH (2018, Egypt, diabetic subgroup), Barwijuk et al. (2024, Poland, single-blind, placebo-controlled), Thanachaiviwat et al. (2024, Thailand, cord blood PRP), and Brezeanu et al. (2025, Romania, two complementary studies: a randomized controlled pilot and a prospective observational arm) [16,17,18,19,20,21,22,23]. Risk of bias was independently assessed using the Cochrane RoB 2.0 tool for all RCTs and the ROBINS-I instrument for the observational study; the overall certainty of evidence was rated using the GRADE framework [24,25,26,27]. Importantly, PRP is not a single, standardized intervention but a family of biologically distinct products (autologous, leukocyte-poor, and cord blood preparations) delivered via different routes and timings; pooled estimates should therefore be interpreted as an average effect across heterogeneous biological agents rather than as the effect of a single defined therapy.

Across these five RCTs, intraoperative PRP appears to consistently and significantly improve scar quality as rated by patients (POSAS-patient SMD −0.50, *p* = 0.003, I^2^ = 0%) and clinicians (POSAS-observer SMD −0.42, *p* = 0.012, I^2^ = 32%) and reduces early wound inflammation (REEDA SMD −0.52, *p* = 0.002, I^2^ = 0%) [20,21,22]. The POSAS-patient result is particularly stable: two studies with different populations, PRP preparations, and follow-up durations [20,22] produce nearly identical directional estimates, and neither arm shows wound dehiscence. According to GRADE, while PRP shows consistent benefits in scar quality, overall certainty remains limited by small sample sizes and heterogeneity, underscoring the need for adequately powered multicenter trials [26].

Three nuances must be front and center. First, VAS pain intensity at early timepoints is not significantly reduced (SMD −0.22, *p* = 0.12, I^2^ = 0%) [20,21,22]. This result is robust and clinically coherent: PRP accelerates structural tissue reorganization in a way that becomes detectable as a scar quality difference at 6–12 weeks but does not blunt the acute nociceptive signal in the recovery room [9,10]. Analgesic consumption data from Barwijuk 2024 (fewer paracetamol doses, *p* = 0.006) and Tehranian 2016 (93% vs. 79% cumulative VAS reduction at 8 weeks, *p* < 0.001) suggest a delayed analgesic effect not captured by early VAS scores [17,20]. Second, VSS heterogeneity is extreme (I^2^ = 94%), driven by the very large CB-PRP effect (Thanachaiviwat 2024, SMD −1.97) versus the smaller LP-PRP effect (Brezeanu 2025, SMD −0.31) [20,21,22]. These cannot be pooled meaningfully; a head-to-head trial is required. Third, REEDA pooling is conditional on correct identification of the dispersion measure in Thanachaiviwat 2024, a methodological detail with substantial consequences: the naive SD interpretation yields I^2^ = 98%, precluding pooling [21]. Because the pooled REEDA estimate is contingent on interpreting the Thanachaiviwat 2024 dispersion measures as standard errors, this outcome should be regarded as the least robust of our primary findings [21]. Although the back-calculation strongly supports the SEM interpretation (full derivation in Supplementary Table S1), this remains an inference rather than a confirmation from the original authors, and the REEDA result must be interpreted with corresponding caution.

Given that two studies included in the quantitative synthesis share authorship with the present review team, particular attention was paid to minimizing potential bias. RoB assessment for these studies was performed by an independent external reviewer using validated instruments [22,23,24,25], and pre-specified sensitivity analyses were conducted to evaluate their influence on pooled estimates. The direction and magnitude of effect for POSAS and REEDA remained consistent with and without the inclusion of these studies, suggesting that overall conclusions are not driven by author-affiliated data alone. Nevertheless, their proportional contribution to a small evidence base must be acknowledged when interpreting the robustness of pooled estimates. Specifically, the author-affiliated RCT (Brezeanu 2025 S1) contributed 61.5% of the weight to the POSAS-patient pool, 58.8% to POSAS-observer, and 60.2% to REEDA [22]. When this study was removed in the pre-specified leave-one-out analysis, the direction of effect was preserved for all outcomes, although only one study then remained per outcome, precluding pooled estimation; this reinforces that our pooled findings are not independent of author-affiliated data and require external confirmation.

### 4.2. The Impact of the Elkhouly Retraction

The formal retraction of Elkhouly et al. (2021) by Karger Publishers (PMID 40435965, May 2025) materially affects the evidence landscape for this field. This study, enrolling 200 patients with substantial reported benefits across REEDA, VSS, and VAS, was one of the most-cited publications in this area and influenced prior narrative reviews and clinical discussions. The retraction, following an Expression of Concern regarding data integrity, removes a large dataset from the evidence pool. Crucially, the related concurrent review (Sen et al., INPLASY202640041, October 2025) explicitly excluded this study, an important step and both reviews reach consistent conclusions regarding the benefit of PRP on scar quality [15]. Earlier reviews or clinical commentaries that incorporated Elkhouly without acknowledging the retraction should be treated with appropriate caution regarding their pooled effect estimates.

### 4.3. Relationship to the Concurrent Systematic Review (Sen et al., 2025 [15])

A related systematic review (Sen S and Gulel Sen F, INPLASY202640041, doi: 10.37766/inplasy2026.4.0041, October 2025) was identified during PROSPERO registration and is cited transparently [15]. Both reviews analyze the same five core RCTs [17,19,20,21,22] and reach consistent primary conclusions: PRP improves scar quality (POSAS) and early wound healing (REEDA); VAS pain is not significantly reduced. The key methodological contribution shared by both reviews is the SEM/SD identification for Thanachaiviwat 2024 [21].

The present review extends and complements Sen et al. in several respects: prospective PROSPERO registration (CRD420261383413) per PRISMA 2020 versus INPLASY only [15,16]; explicit documentation and impact analysis of the Elkhouly retraction; inclusion of the Kamel 2018 [19] diabetic-population subgroup, absent from Sen et al. [15]; inclusion of MSS and NRS as outcome domains for the first time in this field, from Brezeanu 2025 [22,23]; first-hand granular data from the multidimensional Brezeanu 2025 biphasic LP-PRP protocol [22,23]; and uterine scar analysis incorporating the Chaichian 2022 re-analysis [18].

### 4.4. The Uterine Scar: An Emerging Priority

This review confirms that the uterine scar is the most clinically consequential and the least studied dimension of post-cesarean wound healing. Between one-third and two-thirds of women develop a uterine niche (isthmocele) within the first year after a first cesarean section, with profound implications for subsequent reproductive outcomes including abnormal uterine bleeding, infertility, ectopic implantation, and placenta accreta spectrum [8,28]. A recent systematic review by Debras et al. comprehensively catalogued the determinants of uterine wound healing and identified closure technique, suture material, and tissue handling as key modifiable factors; biological adjuncts at the uterotomy site represent a logical but understudied extension of this framework [28].

Chaichian 2022 is the only RCT to evaluate the uterine scar as a primary endpoint [18]. Niche formation was 2/15 (13.3%) in the PRP group versus 7/15 (46.7%) in controls; RR = 0.29 (95% CI 0.07–1.16); and Fisher *p* = 0.109 [18]. The study-reported *p* = 0.002 cannot be reproduced from the published 2 × 2 table and almost certainly derives from a continuous endpoint (niche depth or residual myometrial thickness) rather than the binary rate. The binary result does not reach conventional statistical significance; however, the absolute difference of 33 percentage points is clinically meaningful and directionally consistent with the biological hypothesis. This finding must be regarded as strictly exploratory and hypothesis generating: it rests on a single underpowered trial (*n* = 15 per group) whose 95% confidence interval (RR 0.29, 0.07–1.16) crosses the null and whose reported *p*-value could not be reconciled with the published binary data. No clinical inference regarding niche prevention can or should be drawn at this stage. The field requires an adequately powered multicenter RCT (estimated *n* > 200 per arm) with pre-specified continuous ultrasound niche parameters as the primary endpoint, following the methodological recommendations for uterine scar studies established by Debras et al. [28].

### 4.5. CB-PRP Versus Autologous PRP: A Critical Knowledge Gap

The Thanachaiviwat 2024 CB-PRP data and the Brezeanu 2025 LP-PRP data cannot be pooled for VSS (I^2^ = 94%) [21,22]. The magnitude difference (SMD −1.97 vs. −0.31) may reflect genuine biological superiority of CB-PRP due to a higher fetal platelet growth factor payload or may reflect different assessment timepoints (week 8 vs. day 40) and populations [14,22]. CB-PRP offers a logistical advantage unique to the cesarean setting: cord blood is routinely collected at delivery, requiring no additional maternal phlebotomy [21]. More broadly, PRP represents one of several autologous biological adjuncts under investigation in obstetric wound healing; complementary approaches including hydrogel-based growth factor delivery systems studied in the episiotomy context suggest that the optimal strategy may ultimately involve combined biological scaffolding [28,29]. The only way to definitively compare CB-PRP, LP-PRP, and standard autologous PRP is a three-arm RCT; that trial has not yet been conducted.

### 4.6. The Population Problem

Three out of five RCTs excluded women with diabetes, BMI > 35–40, or emergency CS, exactly the patients at highest absolute risk of wound breakdown [5]. Tehranian 2016, which enrolled this high-risk population, produced the most dramatic REEDA improvement and is the only study with a clinically meaningful baseline complication rate against which to evaluate any intervention [17]. Demonstrating PRP efficacy in healthy young women undergoing elective CS for breech presentation, while scientifically valid, does not address the clinical priority. Future trials must specifically target women with obesity, diabetes, and multiple prior cesarean sections [5,17].

### 4.7. Clinical Interpretation

From a clinical perspective, the findings of this meta-analysis suggest that intraoperative PRP may represent a useful adjunct for improving postoperative scar quality after cesarean section. The random-effects meta-analytic model employed and the Hedges’ g standardized effect size metric were specifically selected to account for expected between-study heterogeneity arising from differences in PRP preparation, activation status, and administration route factors that limit direct comparison but do not negate the consistent directional signal observed across studies [30,31,32].

The most consistent benefit was observed in patient-reported outcomes (POSAS), indicating that PRP positively influences patient perception of scar appearance and healing an aspect increasingly recognized as clinically relevant in modern obstetric care [6,7,20,22]. The improvement in early wound healing (REEDA) further supports the biological plausibility of PRP in modulating the inflammatory and proliferative phases of tissue repair [9,10,21,22]. However, this effect should be interpreted with caution given the limited number of contributing studies and methodological variability across protocols [30]. Regarding the clinical meaningfulness of these effects, the pooled SMDs observed (−0.42 to −0.52) correspond to small-to-moderate effects by Cohen’s conventions. Whether they translate into patient-perceptible benefit remains uncertain, as no validated minimal clinically important difference (MCID) has been established for POSAS or REEDA in the post-cesarean setting. Applying the POSAS MCID proposed in the burn scar literature (approximately 8–10 points on the total scale), the absolute between-group differences in the contributing trials approached but did not consistently exceed this threshold. The observed improvements should therefore be interpreted as biologically plausible and statistically consistent but of as-yet-unconfirmed clinical magnitude; future trials should pre-specify MCID-anchored endpoints and patient-reported cosmetic satisfaction.

Importantly, PRP did not demonstrate a significant reduction in early postoperative pain intensity [20,21,22]. This suggests that its primary mechanism of action relates to structural tissue regeneration rather than acute nociceptive modulation [9]. Clinicians should therefore not expect immediate analgesic benefits following PRP application, though reduced analgesic consumption at later timepoints warrants further investigation [20]. A further source of clinical heterogeneity concerns the differing scar-assessment timepoints: day 40 (Brezeanu 2025) versus day 90 (Barwijuk 2024) [20,22]. Cutaneous scar remodeling is dynamic: by day 40 the scar remains in the early proliferative-to-early-remodeling phase, whereas by day 90 it has entered a more mature remodeling phase with greater collagen reorganization. The consistent directional benefit at both timepoints suggests a PRP effect that is detectable early and persists into later maturation; however, the larger effect at day 90 (SMD −0.73) versus day 40 (SMD −0.39) may reflect either progressive divergence of scar trajectories or between-study differences in PRP preparation. Standardized assessment timepoints (6 weeks, 3 months, and 6 months) are needed in future trials to characterize the temporal profile of any PRP benefit.

The potential effect on uterine scar healing, particularly in reducing niche formation, represents a clinically meaningful but exploratory finding [19]. If confirmed in adequately powered studies, this could have significant long-term implications for reproductive outcomes [8,29]. From a safety perspective, no PRP-related adverse events were reported across any included study, supporting its favorable safety profile [13,18,19,20,21,22,23,24,25]. However, given the small sample sizes, short follow-up, and absence of systematic safety surveillance, these data are insufficient to establish the safety of PRP; they indicate only that no harms were detected within the limited evidence available [13,17,18,19,20,21,22,23,24].

Overall, PRP should not yet be considered standard of care in cesarean section. However, it may be a reasonable option in selected patients at higher risk of impaired wound healing, where even modest improvements in healing outcomes may be clinically meaningful.

### 4.8. Limitations

This review has several important limitations. First, the primary meta-analyses for POSAS and REEDA each pool only two studies (fewer than 150 participants per outcome). This small evidence base limits statistical power, widens confidence intervals, and renders the pooled estimates vulnerable to the influence of any single study; the results should therefore be regarded as preliminary rather than definitive [20,21,22]. Second, PRP protocols remain highly heterogeneous across studies centrifuge speed, spin duration, activation status, volume, and administration route all vary limiting interpretability per standard meta-analytic guidance [17,18,19,20,21,22,23,29]. Third, maximum follow-up is 90 days in most studies [20]; long-term scar outcomes and uterine niche data are limited to a single small trial [18]. Fourth, Brezeanu 2025 studies share authorship with the review team, a declared COI mitigated through independent RoB 2.0 and ROBINS-I assessment and sensitivity analyses [22,23,24,25,26]. Fifth, Elhelw 2025 could not be fully characterized (full-text access pending) [24]. Sixth, fewer than 5 studies per outcome prevents formal publication bias assessment per Cochrane recommendations [24]. Seventh, the DerSimonian–Laird model and Hedges’ g were applied as pre-specified statistical approaches; while appropriate for this evidence base, their use with only two studies per pool limits the stability of heterogeneity estimates [31,32]. Finally, the related Sen et al. (2025) review was identified, but full-text access for exhaustive methodological comparison was limited within the submission timeline [15].

### 4.9. Future Research Priorities

Given WHO projections of cesarean section rates reaching 28.5% globally by 2030, the need for scalable, evidence-based wound management strategies will intensify considerably [33]. Based on the gaps identified in this review, the following priorities are recommended:

1. Multicenter RCT in high-risk populations (obesity, diabetes, and emergency/repeat CS) with *n* ≥ 150/arm [5,18,33].
2. Head-to-head three-arm comparison: CB-PRP [21] vs. LP-PRP [22] vs. standard autologous PRP, with standardized growth factor characterization.
3. Long-term follow-up (12–24 months) with transvaginal ultrasound niche assessment as primary endpoint, incorporating the methodological framework established for uterine scar studies [18,28].
4. Standardized PRP characterization reporting (platelet count, growth factor concentrations, leukocyte content, and activation protocol) in all future publications, per Cochrane Handbook guidance [31].
5. Patient-reported outcome measures beyond scar appearance: quality of life, sexual satisfaction, and body image [6,7].
6. Investigation of combined biological adjunct strategies—including scaffold-based growth factor delivery approaches [29]—alongside PRP in a factorial design.
7. Health economic evaluation of routine intraoperative PRP across risk strata [33].

## 5. Conclusions

This meta-analysis of five randomized controlled trials (*n* = 366) indicates that intraoperative PRP is associated with improved post-cesarean scar quality (POSAS-patient SMD −0.50; observer −0.42) and reduced early wound inflammation (REEDA −0.52), with a reassuring safety profile and no reported adverse events. While early postoperative pain was not significantly reduced, the consistent benefit across patient and clinician-reported scar outcomes, observed despite differing PRP preparations and assessment timepoints, supports the biological plausibility of PRP as a regenerative adjunct in cesarean surgery. These encouraging findings, derived from a still limited and heterogeneous evidence base, should be interpreted as a promising foundation rather than a definitive conclusion. Adequately powered multicenter trials, employing standardized PRP protocols, comparing subtypes directly, and incorporating long-term and uterine scar outcomes, will be valuable to confirm and extend these results and to define the role of PRP in routine obstetric practice.

## Figures and Tables

**Table 1:** Characteristics of all included studies (*n* = 10).

No.	Author, Year	Country	Design	*n* (PRP/Ctrl)	PRP-Type	Activation	Route	Key Scales	Follow-Up	Synthesis
1	Tehranian et al., 2016 [17]	Iran	RCT	138 (67/71)	Autologous (standard)	Not activated	Wound surface + subcut.	REEDA VSS VAS	5 days 8 weeks	Narrative (VSS/REEDA) + primary VAS
2	Chaichian et al., 2022 [18]	Iran	RCT Double-blind	30 (15/15)	Autologous (standard)	Not activated	Intramyometrial (decidua- myometrium)	Niche rate RMT (ultrasound)	Day 1 6 months	Uterine outcomes only
3	Kamel HH, 2018 [19]	Egypt	RCT	120 (60/60)	Autologous (standard)	Not activated	Subcutaneous	REEDA VAS	NR	Subgroup (diabetic pop.)
4	Barwijuk et al., 2024 [20]	Poland	RCT Single-blind	46 (23/23)	Autologous (aPRP; PDGF-AB 140 ng/mL)	Activated	Fascial + subcutaneous micro-inject.	POSAS (P + O) VAS	Days 8, 30, 90	Primary meta-analysis
5	Thanachai- viwat 2024 [21]	Thailand	RCT	52 (26/26)	CB-PRP (cord blood 2.5×)	Activated	Subcutaneous (4 mL) + wound (1 mL)	REEDA VSS VAS †	Days 1, 3 8 weeks	Primary meta-analysis
6	Brezeanu et al., 2025 (S1-RCT) [22]	Romania	RCT Single-blind	100 (50/50)	LP-PRP (leukocyte- poor)	Not activated	Bifasic intraop: uterine margins (5 mL) + subcut. (5 mL)	REEDA POSAS (P + O) VSS VAS, NRS MSS	Day 7 Day 40	Primary meta-analysis
7	Brezeanu et al., 2025 (S2-Obs.) [23]	Romania	Prosp. Observ. (no ctrl)	50 (single arm)	LP-PRP (leukocyte- poor)	Not activated	Bifasic intraop: uterine margins + subcutaneous	REEDA POSAS (P + O) VSS VAS, NRS MSS CBC	Day 7 Day 40	Narrative; ROBINS-I
8	Elhelw et al., 2025 [24]	NR	RCT (confirm.)	NR	Autologous	NR	Local	REEDA POSAS VAS	2 weeks	Under review
9	NCT03602950 (unpubl.)	NR	RCT (registered)	NR	Autologous	NR	Local	REEDA VAS	NR	PI contact pending
10	NCT03497325 (Ain Shams)	Egypt	RCT Ph2	110 (55/55)	Autologous	NR	Intramyom- etrial	RMT (ultrasound)	NR	Uterine narrative only

RCT = randomized controlled trial; Obs. = observational; LP-PRP = leukocyte-poor PRP; CB-PRP = cord blood PRP; aPRP = activated PRP; REEDA = Redness Edema Ecchymosis Discharge Approximation; POSAS = Patient and Observer Scar Assessment Scale (P = patient; O = observer); VSS = Vancouver Scar Scale; MSS = Manchester Scar Scale; VAS = Visual Analogue Scale; NRS = Numeric Rating Scale; RMT = residual myometrial thickness; CBC = complete blood count; NR = not reported; † Thanachaiviwat 2024: dispersion values for REEDA and VAS reported as SEM, not SD (verified by *p*-value back-calculation) [21]; bifasic intraop = application along uterine incision margins before hysterorrhaphy plus subcutaneous layer before skin closure.

**Table 2:** Risk-of-bias assessment: Cochrane RoB 2.0 (RCTs) and ROBINS-I (observational).

Study	D1 Randomization	D2 Deviations	D3 Missing Data	D4 Measurement	D5 Reporting	Overall
Tehranian 2016 [17]	Low	Low	Low	Low	Low	LOW
Chaichian 2022 [18]	Some concerns (alloc. method not described)	Low	Some concerns (6 mo loss not reported)	Low	Some concerns (*p*-value inconsistency *)	SOME CONCERNS
Kamel HH 2018 [19]	Some concerns	Low	Low	Low	Low	SOME CONCERNS
Barwijuk 2024 [20]	Low	Low	Low	Low	Low	LOW
Thanachaiviwat 2024 [21]	Low	Low	Low	Some concerns (SEM labeling, biological data not in question)	Low	LOW
Brezeanu 2025 S1 (RCT) [22]	Low	Low	Low	Some concerns † (assessors not blinded; statistician was)	Low	SOME CONCERNS †
Brezeanu 2025 S2 (ROBINS-I) [23]	Moderate (no random.)	Moderate	Low	Moderate	Low	MODERATE RISK

D1–D5 = RoB 2.0 domains. * Chaichian 2022: study-reported *p* = 0.002 for binary niche rate cannot be reproduced from the published 2 × 2 table (Fisher exact *p* = 0.109) [18]; likely reflects a continuous measure (niche depth/RMT). † Brezeanu 2025 S1 and S2: RoB assessed by an independent external reviewer due to shared authorship with review team [22,23].

**Table 3:** Within-group scar and pain outcomes, Brezeanu 2025 Study 2 (observational, *n* = 50, LP-PRP) [23].

Scale	Day 7 (Mean ± SD)	Day 40 (Mean ± SD)	*p*-Value (Wilcoxon)
Manchester Scar Scale (MSS)	9.2 ± 1.8	6.7 ± 1.5	<0.001
POSAS Total	32.5 ± 6.0	21.4 ± 4.3	<0.001
Vancouver Scar Scale (VSS)	8.1 ± 2.1	5.2 ± 1.4	<0.01
REEDA Scale	7.8 ± 2.0	3.4 ± 1.1	<0.001
VAS (pain)	6.0 ± 1.5	2.0 ± 0.9	<0.001
NRS (pain)	5.0 ± 1.4	1.0 ± 0.8	<0.001
Overall composite score	8.88 ± 2.13	6.46 ± 1.23	<0.001

All six scar and pain scales improved significantly between Day 7 and Day 40 (Wilcoxon signed-rank test). Hematological parameters: hemoglobin and hematocrit increased significantly by Day 40 (*p* < 0.01), consistent with postoperative recovery; leukocyte count decreased significantly (*p* < 0.01), consistent with resolution of postoperative inflammation [23].

**Table 4:** Extracted outcome data from all contributing studies (mean ± SD unless stated).

Study	Outcome	Timepoint	Disp. Unit	PRP (Mean±)	Control (Mean±)	*p*	SMD (95% CI)
Barwijuk 2024 (*n* = 23/23) [20]	POSAS-patient	Day 8	SD	16.96 ± 1.52	17.57 ± 1.85	0.230	−0.36 (−0.94, +0.22)
	POSAS-patient	Day 30	SD	17.00 ± 1.76	18.09 ± 2.00	0.033 *	−0.58 (−1.18, +0.02)
	POSAS-patient	Day 90	SD	14.91 ± 1.54	16.09 ± 1.68	0.021 *	−0.73 (−1.33, −0.13)
	POSAS-observer	Day 90	SD	13.39 ± 1.53	14.74 ± 2.11	0.002 *	−0.73 (−1.33, −0.13)
	VAS	0 h	SD	3.30 ± 2.23	3.96 ± 2.08	0.299	−0.31 (−0.89, +0.28)
	VAS	6 h	SD	4.96 ± 1.49	5.30 ± 1.52	0.465	−0.23 (−0.81, +0.36)
Thanachaiviwat 2024 (*n* = 26/26) [21]	REEDA	Day 1	SEM †	1.50 ± 0.256	2.50 ± 0.267	0.009 *	−0.75 (−1.31, −0.19)
	REEDA	Day 3	SEM †	0.92 ± 2.60	1.12 ± 2.17	0.572	−0.08 (−0.63, +0.47)
	VSS	Week 8	SD	2.58 ± 2.00	6.96 ± 2.44	<0.001 *	−1.97 (−2.63, −1.30)
	VAS	Day 1	SEM †	3.73 ± 0.439	4.19 ± 0.464	0.473	−0.20 (−0.74, +0.35)
Chaichian 2022 (*n* = 15/15) [18]	Niche rate	6 months	binary	2/15 (13.3%)	7/15 (46.7%)	Fisher *p* = 0.109 ‡	RR 0.29 (0.07, 1.16)
Brezeanu 2025 S1 (*n* = 50/50) [22]	POSAS-patient	Day 7	SD	10.08 ± 3.28	11.00 ± 4.00	0.231	−0.25 (−0.65, +0.14)
	POSAS-patient	Day 40	SD	7.24 ± 1.81	8.00 ± 2.06	0.029 *	−0.39 (−0.79, +0.00)
	POSAS-observer	Day 40	SD	6.46 ± 1.23	6.84 ± 1.39	0.300	−0.29 (−0.68, +0.10)
	REEDA	Day 7	SD	0.88 ± 1.09	1.56 ± 2.10	NS	−0.41 (−0.80, −0.01)
	VSS	Day 40	SD	0.46 ± 0.73	0.76 ± 1.18	NS	−0.31 (−0.70, +0.09)
	VAS	Day 7	SD	1.08 ± 0.89	1.30 ± 1.32	NS	−0.20 (−0.59, +0.19)
	NRS	Day 40	SD	0.10 ± 0.36	0.60 ± 0.80	NS	−0.81 (−1.21, −0.40)

* *p* < 0.05. † SEM: reported as standard error of the mean; converted to SD (SD = SEM × √26) for analysis; verified by *p*-value back-calculation. Sensitivity using naive SD: REEDA I^2^ = 98%, pooling inappropriate. ‡ Fisher’s exact *p* from 2 × 2 table; study-reported *p* = 0.002 inconsistent with binary niche rate, likely reflects continuous endpoint. NS = not significant (exact *p* not given).

**Table 5:** Summary of Findings (GRADE).

Outcome	Effect Estimate	Participants (Studies)	Certainty of Evidence (GRADE)	Comments
Scar quality (POSAS—patient)	SMD −0.50 (95% CI −0.83 to −0.17)	146 (2 RCTs)	Moderate	Consistent effect, low heterogeneity
Scar quality (POSAS—observer	SMD −0.42 (95% CI −0.75 to −0.10)	146 (2 RCTs)	Moderate	Some risk of bias due to lack of blinding
Early wound healing (REEDA)	SMD −0.52 (95% CI −0.84 to −0.20)	102 (2 RCTs)	Low to Moderate	Imprecision and SEM/SD ambiguity in one study
Postoperative pain (VAS)	SMD −0.22 (95% CI −0.50 to +0.06)	198 (3 RCTs)	Moderate	No significant effect; consistent findings
Scar quality (VSS)	Not pooled (I^2^ = 94%)	2 RCTs	Low	High heterogeneity; different PRP types
Uterine scar (niche formation)	RR 0.29 (95% CI 0.07–1.16)	30 (1 RCT)	Low	Underpowered; inconsistent reporting
Adverse events	None reported	All Studies	Low to Moderate	Limited sample size; possible under-reporting

Footnotes (GRADE Justification): Risk of bias: Downgraded by one level due to some concerns in included RCTs, particularly related to lack of blinding of outcome assessors and incomplete reporting of allocation procedures in certain studies. Imprecision: Downgraded by one level due to small sample sizes and limited number of studies contributing to pooled estimates (typically ≤2 RCTs per outcome), resulting in wide confidence intervals. Inconsistency: Downgraded by one level when substantial heterogeneity was present (I^2^ ≥ 75%), as observed for VSS outcomes, precluding meaningful meta-analysis. Indirectness: Downgraded by one level due to variability in intervention protocols (PRP preparation methods, activation status, and administration routes) and differences in outcome assessment timepoints across studies. Publication bias: Not formally assessed due to the limited number of studies (<5 per outcome), which precludes reliable funnel plot analysis. Uterine scar outcomes: Downgraded by two levels due to serious imprecision (single small RCT, *n* = 30) and inconsistency in reported statistical results (discrepancy between reported and recalculated *p*-values). Adverse events: Downgraded by one level due to imprecision and potential under-reporting, as safety outcomes were not systematically or uniformly assessed across studies. REEDA outcome: Downgraded by one level due to methodological uncertainty related to dispersion measures (SEM vs. SD) in one contributing study, although sensitivity analyses support the primary interpretation.

## Data Availability

All data are included in the manuscript and Supplementary Materials. The search strategy, full data extraction forms, and PRISMA checklist are available on the PROSPERO record (CRD420261383413) and upon request from the corresponding author.

## Supplementary Materials

The following supporting information can be downloaded at: https://www.mdpi.com/article/10.3390/healthcare14142108/s1.

## Abbreviations

The following abbreviations are used in this manuscript:

CS	Cesarean section
PRP	Platelet-rich plasma
PDGF	Platelet-derived growth factor
TGF-β1/2	Transforming growth factor-beta 1 and 2
VEGF	Vascular endothelial growth factor
EGF	Epidermal growth factor
FGF	Fibroblast growth factor
IGF 1/2	Insulin-like growth factor-1 and -2
CB-PRP	Cord blood PRP
REEDA	Redness, Edema, Ecchymosis, Discharge, Approximation
POSAS	Patient and Observer Scar Assessment Scale
VSS	Vancouver Scar Scale
MSS	Manchester Scar Scale
VAS	Visual Analogue Scale
NRS	Numeric Rating Scale
LP-PRP	Leukocyte-poor platelet-rich plasma
RCT	Randomized controlled trial
SMD	Standardized mean difference
CI	Confidence interval
RR	Risk ratio
GRADE	Grading of Recommendations Assessment, Development and Evaluation
RoB	Risk of bias
ROBINS-I	Risk Of Bias In Non-randomized Studies of Interventions
PRISMA	Preferred Reporting Items for Systematic Reviews and Meta-Analyses
PROSPERO	International Prospective Register of Systematic Reviews
CBC	Complete blood count
RMT	Residual myometrial thickness
