# Supplementary material for: Platelet-Rich Plasma for Wound Healing and Scar Outcomes After Cesarean Section: A Systematic Review and Meta-Analysis
Source: Healthcare (Basel). 2026 Jul 14;14(14):2108. doi: 10.3390/healthcare14142108 (PMC13411088; doi:10.3390/healthcare14142108)
Supplement: Supplementary file 1 [file healthcare-14-02108-s001.zip › healthcare-4328088-supplementary.pdf]

Ok PRISMA 2020 CHECKLIST

Platelet-Rich Plasma for Wound Healing and Scar Outcomes After Cesarean Section: A Systematic Review and Meta-Analysis

**Manuscript ID**	healthcare-4328088
**Journal**	Healthcare (MDPI) | ISSN 2227-9032
**Authors**	Brezeanu AM; Brezeanu D; Tica V-I
**Affiliation**	Ovidius University of Constanta, Romania
**PROSPERO**	CRD420261383413 (registered April 2026)
**PRISMA version**	PRISMA 2020 — Page MJ et al., BMJ 2021;372:n71
**Checklist completed**	May 2026
**Flow diagram**	Included as Figure 1 in the manuscript

#	PRISMA 2020 Item	How addressed in the manuscript	Location
**TITLE**			
1	**Title**	The title explicitly identifies the report as a systematic review and meta-analysis: "Platelet-Rich Plasma for Wound Healing and Scar Outcomes After Cesarean Section: A Systematic Review and Meta-Analysis"	**Title page**
**ABSTRACT**			
2	**Abstract**	Structured abstract with labeled sections: Background, Objectives, Methods, Results, Conclusions. Includes: PROSPERO registration (CRD420261383413), PRISMA 2020 compliance, databases searched, eligibility criteria, number of included studies (5 RCTs, n=366), all primary and secondary outcome results with effect sizes (SMD, 95% CI, I^2), GRADE certainty ratings, and conclusions.	**Abstract (pp. 1–2)**
**INTRODUCTION**			
3	**Rationale**	Section 1 (Background) describes: (1) global CS rates and associated wound morbidity; (2) biological rationale for PRP (growth factor mechanisms); (3) the formal retraction of Elkhouly et al. 2021 (PMID 40435965) and its impact on prior evidence; (4) gaps not addressed by existing literature including the concurrent Sen et al. (2025) review.	**Section 1 (Background)**
4	**Objectives**	Section 1 concludes with four explicit objectives: (1) synthesize RCT evidence using rigorous meta-analytic methods including dispersion-measure verification; (2) address the confirmed retraction; (3) explore uterine scar as a distinct outcome domain; (4) provide GRADE-rated evidence summary. The PICO question is stated: population (adult women undergoing CS), intervention (PRP any type), comparator (standard care/placebo),	**Section 1 (Background)**

#	PRISMA 2020 Item	How addressed in the manuscript	Location
		outcomes (REEDA, POSAS, VSS, MSS, VAS/NRS, uterine scar, complications).	
**METHODS**			
5	**Eligibility criteria**	Section 2.1 specifies full PICO eligibility criteria:<br>• Population: adult women (≥18 y) undergoing elective or emergency CS<br>• Intervention: PRP any type (autologous, LP-PRP, CB-PRP), any route, any activation status<br>• Comparator: standard care or saline placebo<br>• Outcomes: ≥1 quantifiable wound/scar outcome<br>• Study designs: RCTs (primary); NRCTs and prospective controlled observational (secondary)<br>Exclusion criteria explicitly listed including retracted publications (Elkhouly 2021).	**Section 2.1**
6	**Information sources**	Section 2.2 specifies: PubMed/MEDLINE, Cochrane CENTRAL, Embase, Web of Science Core Collection, Scopus — all searched from inception to April 2026 without date or language restrictions. ClinicalTrials.gov and WHO ICTRP searched for registered trials. Reference lists of all included studies screened manually. Authors of unpublished registered trials (NCT03602950, NCT03497325) contacted directly.	**Section 2.2**
7	**Search strategy**	Section 2.2 presents the core Boolean search string with three conceptual blocks (PRP terminology, cesarean section terms, outcome terms). Full database-specific search strings — including MeSH terms for PubMed, Emtree for Embase, TS= field tags for Web of Science, TITLE-ABS-KEY for Scopus — are available in the supplementary search strategy document submitted to PROSPERO (CRD420261383413).	**Section 2.2 + Supplementary**
8	**Selection process**	Section 2.3: Records managed in Covidence. Two independent reviewers (A-M.B. and D.B.) screened titles/abstracts then full texts. Disagreements resolved by consensus; arbitration by V-I.T. Reasons for exclusion documented per PRISMA requirements and presented in Figure 1 (PRISMA 2020 flow diagram).	**Section 2.3 Figure 1**
9	**Data collection process**	Section 2.4: Data extracted independently by two reviewers using a standardized pre-piloted form. Dispersion-measure labelling verified against reported p-values by back-calculation where ambiguous (Thanachaiviwat 2024: SEM vs SD). Corresponding authors contacted for missing data.	**Section 2.4**
10a	**Data items — outcomes**	Section 2.1 and Table 5 (SoF): Primary outcome = REEDA scale. Secondary outcomes = POSAS (patient + observer), VSS, MSS, VAS, NRS, ultrasonographic scar thickness, wound complication rates, hematological parameters. All outcomes listed with timepoints and measurement instruments.	**Section 2.1 Table 5**
10b	**Data items — other variables**	Table 1 presents for each study: author, year, country, design, sample size per group, PRP type, preparation protocol, activation status, volume,	**Table 1**

#	PRISMA 2020 Item	How addressed in the manuscript	Location
		administration route, assessment timepoints, and synthesis category.	
11	**Study risk of bias assessment**	Section 2.5: Cochrane RoB 2.0 applied to all RCTs across 5 domains (D1–D5). ROBINS-I applied to the observational study (Brezeanu 2025 S2). Due to declared COI, RoB for Brezeanu 2025 studies independently assessed by external reviewer. Results presented in Table 2.	**Section 2.5 Table 2**
12	**Effect measures**	Section 2.6: Continuous outcomes — SMD (Hedges' g) with 95% CI, or MD where same scale used. Dichotomous outcomes — Risk Ratio (RR) with 95% CI. All effect measures pre-specified and applied consistently.	**Section 2.6**
13	**Synthesis methods**	Section 2.6: DerSimonian-Laird random-effects model. Heterogeneity quantified by I^2 and Cochran's Q (substantial threshold I^2>50%). Pre-specified sensitivity analyses: (1) exclude high/some-concerns RoB studies; (2) exclude author-affiliated studies; (3) exclude CB-PRP study; (4) naive SD interpretation for Thanachaiviwat 2024. Pre-specified subgroup analyses: PRP type, activation status, administration route. Analyses: RevMan 5.4 and R (metafor, meta).	**Section 2.6**
14	**Reporting bias assessment**	Section 2.6: Funnel plot asymmetry and Egger's test planned if ≥5 studies contributed to a synthesis. Fewer than 5 studies per outcome precluded formal testing; narrative assessment performed. Selective outcome reporting assessed via RoB 2.0 Domain 5 (comparing registered vs published outcomes). Discussed in Section 4.8 (Limitations).	**Section 2.6 Section 4.8**
15	**Certainty assessment**	Section 2.6: GRADE framework applied for each outcome. Ratings (HIGH/MODERATE/LOW/VERY LOW) based on five domains: RoB, inconsistency, indirectness, imprecision, publication bias. Results presented in Table 5 (Summary of Findings). GRADEpro GDT software used.	**Section 2.6 Table 5**
**RESULTS**			
16	**Study selection**	Section 3.1 describes the full selection process with all numbers at each stage. Figure 1 presents the PRISMA 2020 flow diagram: 109 identified → 62 after deduplication → 17 full texts assessed → 7 excluded with documented reasons (including 1 retracted publication) → 10 qualitative synthesis → 5 RCTs in primary meta-analysis (n=366).	**Section 3.1 Figure 1**
17	**Study characteristics**	Table 1 presents characteristics of all 10 included studies: author, year, country, design, n per group, PRP type, activation status, administration route, scales used, follow-up, and synthesis category. Two corrections from preliminary extraction documented: Tehranian 2016 n=67/71 (not 30/30); Chaichian 2022 = uterine scar (not cutaneous).	**Table 1 Section 3.2**
18	**Risk of bias in studies**	Table 2 presents RoB 2.0 assessments for all 5 RCTs across D1–D5 with overall judgement, and ROBINS-I assessment for the observational study.	**Table 2 Section 3.4**

#	PRISMA 2020 Item	How addressed in the manuscript	Location
		Footnotes explain specific concerns (SEM labelling in Thanachaiviwat; COI in Brezeanu; p-value inconsistency in Chaichian).	
19	**Results of individual studies**	Section 3.4 presents individual study results with means ± SD per group and individual SMDs for all contributing studies. Table 4 presents complete extracted outcome data for all studies including individual effect estimates and 95% CI per study per outcome. Dispersion-measure identification for Thanachaiviwat 2024 documented explicitly (Section 3.3).	**Section 3.4 Table 4**
20	**Results of syntheses**	Sections 3.5.1–3.5.5 and Figures 2–5 present:<br>• POSAS-patient: SMD −0.50 (−0.83, −0.17); p=0.003; I^2=0% (Figure 2)<br>• POSAS-observer: SMD −0.42 (−0.75, −0.10); p=0.012; I^2=32% (Figure 3)<br>• REEDA: SMD −0.52 (−0.84, −0.20); p=0.002; I^2=0% (Figure 4)<br>• VAS: SMD −0.22 (−0.50, +0.06); p=0.12 (NS); I^2=0% (Figure 5)<br>• VSS: I^2=94%; pooling inappropriate — narrative only<br>• Uterine niche (Chaichian 2022): RR 0.29 (0.07, 1.16); Fisher p=0.109<br>Sensitivity analysis results reported for each outcome.	**Section 3.5 Figures 2–5**
21	**Reporting biases**	Fewer than 5 studies per outcome precluded formal funnel plot analysis. Narrative assessment performed (Section 4.8). Selective outcome reporting assessed via RoB 2.0 Domain 5 for each RCT; results shown in Table 2.	**Section 4.8 Table 2**
22	**Certainty of evidence**	Table 5 (Summary of Findings) presents GRADE certainty ratings for all 8 pre-specified outcomes with explicit justification for downgrading. Ratings: MODERATE for POSAS and VAS; LOW–MODERATE for REEDA; LOW for VSS and uterine outcomes; VERY LOW for MSS/NRS (single-arm only).	**Table 5 Section 3.7**
**DISCUSSION**			
23	**Discussion**	Section 4 (Discussion) interprets results in context of existing evidence, including comparison with the concurrent Sen et al. (2025) review (Section 4.3). The clinical interpretation section (4.7) discusses implications for practice. The impact of the Elkhouly 2021 retraction on prior evidence is explicitly discussed (Section 4.2).	**Section 4 (all subsections)**
24	**Limitations**	Section 4.8 describes 8 pre-specified limitations: (1) only 2 studies per outcome pool; (2) heterogeneous PRP protocols; (3) short follow-up (≤90 days); (4) authorship COI for Brezeanu 2025; (5) Elhelw 2025 full-text unavailable; (6) <5 studies prevents publication bias testing; (7) model instability with 2 studies per pool; (8) limited access to Sen et al. 2025 for exhaustive comparison.	**Section 4.8**

#	PRISMA 2020 Item	How addressed in the manuscript	Location
25	**Conclusions**	Section 5 (Conclusions) provides 9 explicit, evidence-grounded conclusions including the negative VAS finding, the CB-PRP vs LP-PRP heterogeneity, the preliminary uterine niche finding, the confirmed retraction, and the recommendation against routine implementation pending adequately powered trials.	**Section 5 (Conclusions)**
**OTHER INFORMATION**			
26	**Registration and protocol**	The review is prospectively registered in PROSPERO: CRD420261383413 (registered April 2026, prior to data extraction). Protocol version 1.0. The full search strategy document was uploaded to PROSPERO. This information is stated in the Abstract and in the Methods (Section 2, opening paragraph).	**Abstract Section 2 PROSPERO**
27	**Support**	No external funding was received. This systematic review received no grant from any public, commercial, or not-for-profit funding agency. Declared in the Funding section of the manuscript.	**Funding section**
28	**Competing interests**	Full conflict of interest declaration provided: two included studies (Brezeanu et al. 2025 S1 and S2, Healthcare) share authorship with the review team. Mitigation strategies declared: independent external RoB assessment; sensitivity analyses with/without author-affiliated studies; transparent reporting of their influence on pooled estimates. No financial ties to PRP device manufacturers.	**COI section**
29	**Availability of data, code and other materials**	All data are contained in the manuscript and tables. Full search strategy available on PROSPERO (CRD420261383413) and on request from the corresponding author. Statistical code available on request.	**Data availability section**

This checklist was completed in accordance with the PRISMA 2020 statement (Page MJ et al., BMJ 2021;372:n71). The PRISMA 2020 flow diagram is included as Figure 1 in the main manuscript.
